# Frequency of expression and generation of T-cell responses against antigens on multiple myeloma cells in patients included in the GMMG-MM5 trial

**DOI:** 10.18632/oncotarget.11215

**Published:** 2016-08-11

**Authors:** Michael Schmitt, Angela G. Hückelhoven, Michael Hundemer, Anita Schmitt, Susanne Lipp, Martina Emde, Hans Salwender, Mathias Hänel, Katja Weisel, Uta Bertsch, Jan Dürig, Anthony D. Ho, Igor Wolfgang Blau, Hartmut Goldschmidt, Anja Seckinger, Dirk Hose

**Affiliations:** ^1^ Universitätsklinikum Heidelberg, Medizinische Klinik V, Heidelberg, Germany; ^2^ Department of Internal Medicine II, Asklepios Klinik Altona, Hamburg, Germany; ^3^ Department of Internal Medicine III, Klinikum Chemnitz GmbH, Chemnitz, Germany; ^4^ Department of Hematology, Oncology and Immunology, University of Tübingen, Tübingen, Germany; ^5^ Department of Hematology, University Hospital Essen, Essen, Germany; ^6^ Medical Clinic III Hematology and Oncology, Charité University Medicine Berlin, Berlin, Germany; ^7^ Nationales Centrum für Tumorerkrankungen, Heidelberg, Germany

**Keywords:** tumor associated antigens, T cells, immunogenicity, multiple myeloma, RNA-sequencing

## Abstract

**Background:**

Raising T-cell response against antigens either expressed on normal and malignant plasma cells (e.g. HM1.24) or aberrantly on myeloma cells only (e.g. cancer testis antigens, CTA) by vaccination is a potential treatment approach for multiple myeloma.

**Results:**

Expression by GEP is found for *HM1.24* in all, *HMMR* in 318/458 (69.4%), *MAGE-A3* in 209/458 (45.6%), *NY-ESO-1/2* in 40/458 (8.7%), and *WT-1* in 4/458 (0.8%) of samples with the pattern being confirmed by RNA-sequencing. T-cell-activation is found in 9/26 (34.6%) of patient samples, i.e. against HM1.24 (4/24), RHAMM-R3 (3/26), RHAMM1-8 (2/14), WT-1 (1/11), NY-ESO-1/2 (1/9), and MAGE-A3 (2/8). In 7/19 T-cell activation responses, myeloma cells lack respective antigen-expression. Expression of *MAGE-A3*, *HMMR* and *NY-ESO-1/2* is associated with adverse survival.

**Experimental design:**

We assessed expression of *HM1.24* and the CTAs *MAGE-A3*, *NY-ESO-1/2*, *WT-1* and *HMMR* in CD138-purified myeloma cell samples of previously untreated myeloma patients in the GMMG-MM5 multicenter-trial by gene expression profiling (GEP; *n* = 458) and RNA-sequencing (*n* = 152) as potential population regarding vaccination trials. We then validated the feasibility to generate T-cell responses (*n* = 72) against these antigens by IFN-γ EliSpot-assay (*n* = 26) related to antigen expression (*n* = 22). Lastly, we assessed survival impact of antigen expression in an independent cohort of 247 patients treated by high-dose therapy and autologous stem cell transplantation.

**Conclusions:**

As T-cell responses can only be raised in a subfraction of patients despite antigen expression, and the number of responses increases with more antigens used, vaccination strategies should assess patients’ antigen expression and use a “cocktail” of peptide vaccines.

## INTRODUCTION

Multiple myeloma is characterized by the accumulation of clonal plasma cells in the bone marrow and associated clinical signs and symptoms, especially those related to the displacement of normal hematopoiesis, generation of osteolytic bone disease, and production of a monoclonal protein [1]. One of the most prominent effects thereof is the induction of immunosuppression visible in dysfunctional dendritic cells (DC) and diminished T-cell activation [2–4]. In turn, immunotherapeutic approaches to eliminate myeloma cells by fostering host immunity are in development. Most advanced is the recruitment of innate immunity to kill myeloma cells by monoclonal antibodies targeting antigens either aberrantly expressed on myeloma cells (e.g. SLAMF7 -elotuzumab [5]) or expressed on both, normal as well as malignant plasma cells (e.g. CD38 -daratumumab [6], isatuximab [7]). A different approach is (re)directing T-cells towards myeloma cell killing. This can be achieved via T-cell genetic engineering with chimeric antigen receptors or recombinant T-cell receptor, both requiring *ex vivo* engineering and expansion of patient specific T-cells [8–11], as well as T-cell bispecific antibodies that simultaneously bind a surface target on tumor cells and an associated T-cell receptor chain present on T-cells thereby inducing potent T-cell mediated killing of cells carrying the target [12, 13]. A further - and potentially even prophylactic - approach is the development of cancer vaccines generating myeloma-specific immunity selectively targeting malignant cells - with limited toxicity to normal tissues [14–16]. Potential targets comprise those constitutively expressed on normal as well as on malignant plasma cells (e.g. HM1.24) [17–19], or those expressed on malignant cells but not their normal counterpart, e.g. cancer testis antigens (CTA). In multiple myeloma, several CTAs have been described by others and us, including MAGE-A3 (melanoma-associated antigen 3) [20–23], NY-ESO-1 (New York esophageal-1) [24–27], WT-1 (Wilms’ tumor gene 1) [28–30], and RHAMM/HMMR (receptor of hyaluronic acid mediated motility) [31–33]. Of these, *MAGE-A3*, *NY-ESO-1/2*, and *WT-1* are not expressed in normal bone marrow plasma cells but aberrantly in malignant plasma cells [34, 35]. *HMMR* [36], *MAGE-A3,* and *NY-ESO-1* [34] are associated with adverse survival.

In this article, we address patients treated by up-front high-dose therapy and autologous stem cell transplantation included in the GMMG-MM5 trial as potential population regarding vaccination trials (*n* = 604). We first assessed expression of HM1.24 and the CTAs HMMR, NY-ESO-1/2, MAGE-A3 and WT-1 by using DNA-microarrays (*n* = 458) and validation by RNA-sequencing (*n* = 152). We next assessed a representative cohort of 72 consecutive patients regarding the possibility to raise T-cell specific answers. Subsequently, the interrelation of antigen expression and generation of T-cell responses was addressed. Lastly, we used a comparable cohort of 247 previously untreated myeloma patients with long-term follow up to investigate the impact of expression of the respective antigens on survival.

## RESULTS

### Antigen-expression

**Figure 1:**
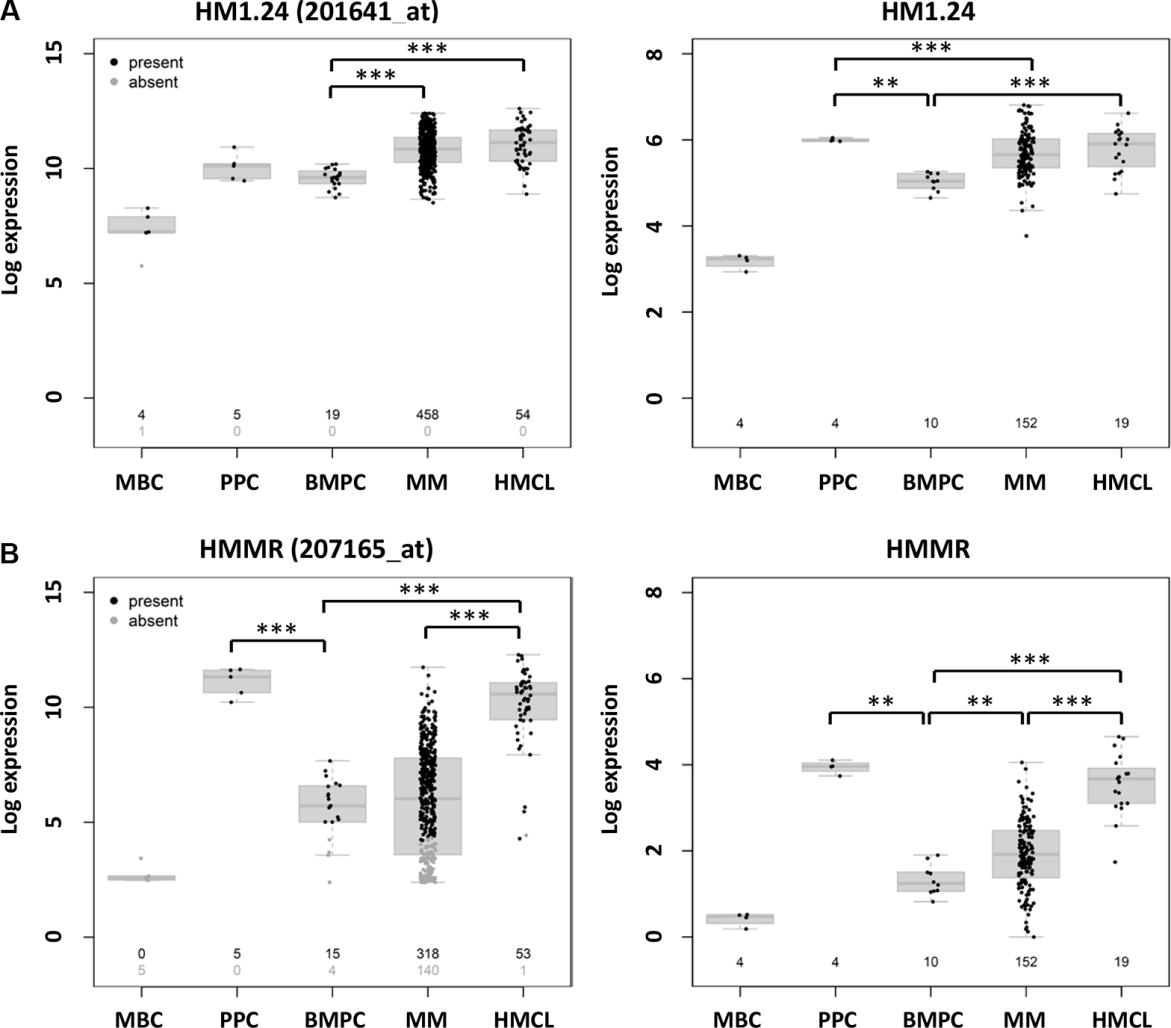

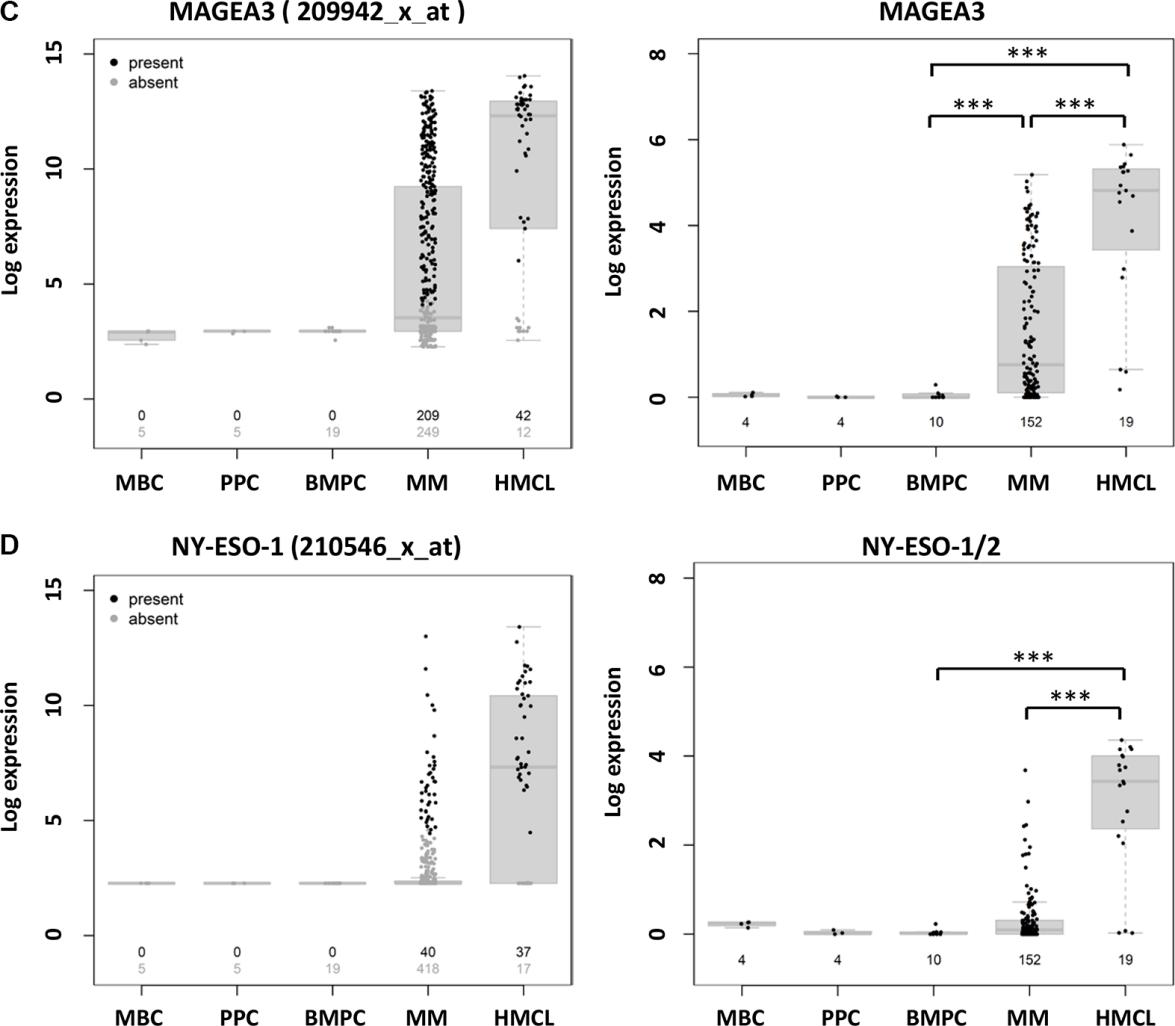

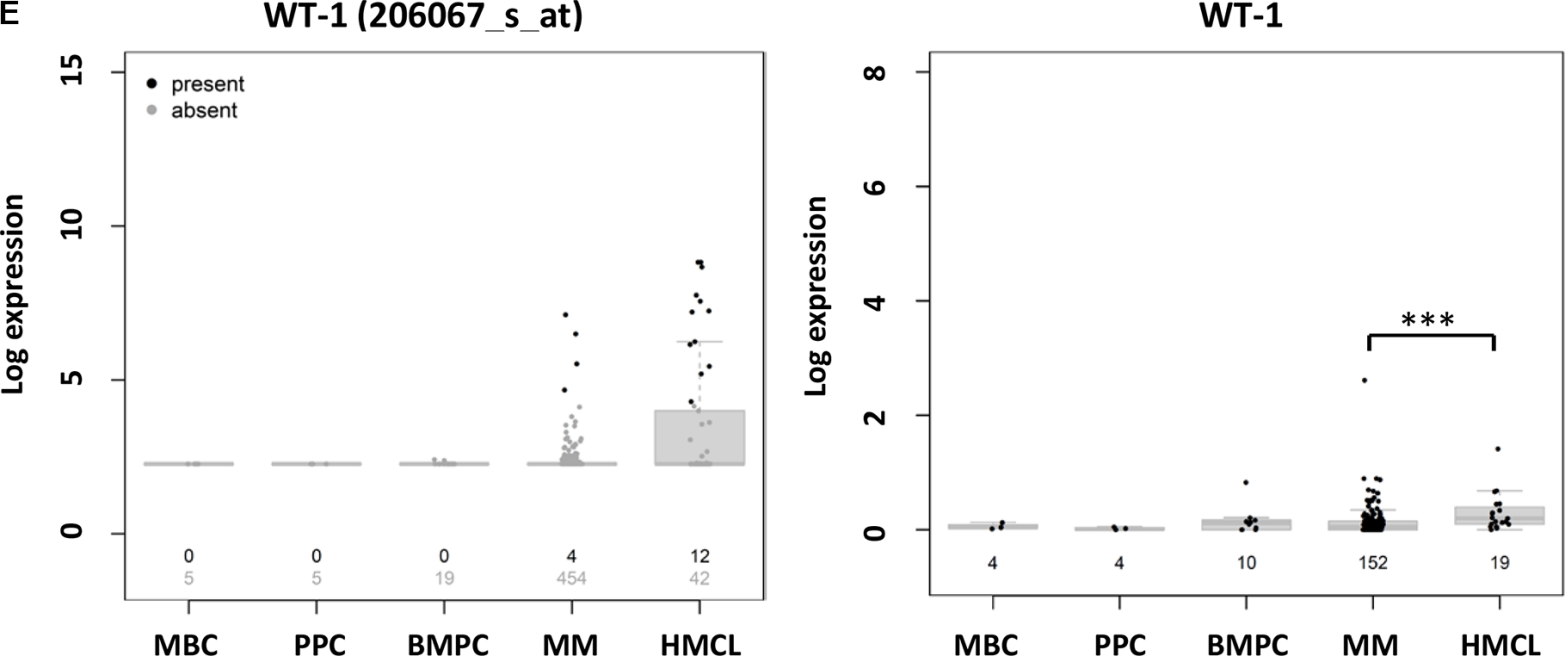
Expression of HM1.24 and cancer testis antigens in normal and malignant plasma cells as well as cells of the B-cell lineage Shown is the expression of (**A**) *HM1.24*, (**B**) *HMMR*, (**C**) *MAGE-A3,* (**D**) *NY-ESO-1/2*, and (**E**) *WT-1* in normal plasma cell precursors, i.e. memory B-cells (MBC) and *in vitro* generated polyclonal plasmablastic cells (PPC), as well as normal bone marrow plasma cells (BMPC), malignant plasma cells from patients with newly-diagnosed multiple myeloma (MM), and human myeloma cell lines (HMCL). Left panel shows gene expression profiling using DNA-microarrays with black numbers depicting the number of patient samples expressing the respective gene according to the PANP-algorithm, gray number the one that showed no expression. Right panel shows gene expression profiling using RNA-sequencing with numbers depicting the total number of samples assessed. Significant difference is depicted by one asterisk (*) for a level of *P* < 0.05, two asterisks (**) for a level of *P* < 0.01, and three (***) for *P* < 0.001.

Antigen expression assessed by DNA-microarrays is depicted in the following for the GMMG-MM5 cohort as well as the part of this cohort validated by RNA-sequencing [in brackets, for gene expression profiling]. HM1.24 is expressed in all 458 CD138-purified plasma cell samples with available gene expression data. *HMMR* is expressed in 318/458 (69.4%) [91.4%] of samples, *MAGE-A3* in 209/458 (45.6%) [46.7%], *NY-ESO-1/2* (NY-ESO1 (CTAG1A, CTAG1B), NY-ESO2 (CTAG2)) in 40/458 (8.7%) [7.2%], and *WT-1* in 4/458 (0.8%) [1.9%] (Figure 1, Table 1, Supplementary Figure S1).

**Table 1:** Expression of HM1.24 and CTAs as well as CTA-specific immune responses

Patient	HM1.24			HMMR-R3	HMMR1-8	HMMR		MAGEA3			NY-ESO-1			WT-1		
#	response	GEP	RNA-seq	response	response	GEP	RNA-seq	response	GEP	RNA-seq	response	GEP	RNA-seq	response	GEP	RNA-seq
1	—	p	—	n	—	a	—	—	p	—	—	a	—	—	a	—
2	++	p	277.56	++	+	p	6.79	n	p	0.07	n	a	0	n	a	0.07
3	—	p	306.18	n	—	p	19.47	—	p	1.42	—	a	0	—	a	17.57
4	++	p	337.21	++	++	p	4.91	n	a	1.41	n	a	0	++	a	0
5	++	p	—	n	++	a	—	—	a	—	n	a	—	n	a	—
6	—	p	—	+	—	p	—	—	p	—	—	a	—	—	a	—
7	n	p	—	n	n	a	—	—	a	—	—	a	—	n	a	—
8	—	p	—	n	—	a	—	—	p	—	—	a	—	—	a	—
9	++	p	—	n	n	a	—	n	a	—	+	a	—	+	a	—
10	—	p	422.29	n	n	p	10.68	—	p	0.48	—	a	0	—	a	0
11	+	p	—	n	n	a	—	n	p	—	n	a	—	n	a	—
12	—	p	460.22	n	—	p	3.95	—	p	0.46	—	a	0	—	a	0
13	+	p	—	n	n	a	—	+	p	—	n	a	—	+	a	—
14	—	—	—	n	—	—	—	—	—	—	—	—	—	—	—	—
15	—	—	—	n	—	—	—	—	—	—	—	—	—	—	—	—
16	—	p	—	n	—	p	—	—	p	—	—	a	—	—	a	—
17	n	—	—	n	n	—	—	n	—	—	n	—	—	n	—	—
18	n	p	243.42	n	n	p	15.17	+	p	37.07	n	a	0.66	+	a	0.2
19	—	p	—	n	—	p	—	—	a	—	—	p	—	—	a	—
20	n	p	226.87	n	n	p	16.63	n	a	0	n	a	0	n	a	0.13
21	n	—	—	n	n	—	—	—	—	—	—	—	—	n	—	—
22	—	p	—	n	n	p	—	—	a	—	—	a	—	—	a	—
23	—	p	—	n	—	p	—	—	p	—	—	a	—	—	a	—
24	n	p	—	n	n	p	—	—	p	—	—	a	—	—	a	—
25	—	p	—	++	—	a	—	—	p	—	—	a	—	—	a	—
26	—	p	—	n	—	p	—	—	p	—	—	a	—	—	a	—

GEP, gene expression profiling, “absence” (a) and “presence” (p) of gene expression assessed by “presence-absence calls with negative-strand matching probe-sets” (PANP) algorithm. RNA-seq, RNA-sequencing, respective quantitative values depicted as normalized count rates. Response: light green (+) indicates specific T-cell response against the respective antigen defined as ≥ 2-fold increased number of positive ELISPOTs compared to control, dark green (++) strong response defined as ≥ 3-fold increased number of positive ELISPOTs compared to control. Orange, response can be raised although the antigen is not expressed. Light gray, no response (n). White, not available.

RNA-sequencing validates expression showing an identical expression pattern for the observed antigens with normalized read counts above one being observed for *HM1.24* in all, *HMMR* in 144/152 (94.7%), *MAGE-A3* in 77/152 (50.7%), *NY-ESO-1/2* in 20/152 (13.2%), and *WT-1* in 5/152 (3.3%) of corresponding samples (Figure 1, Table 1). GEP and RNA-sequencing show an overall correlation (Pearson) for *HM1.24* (*r*^2^ = .68), *HMMR* (*r*^2^ = 72), *MAGE-A3* (*r*^2^ = .74), *NY-ESO-1/2* (*r*^2^ = .72), and *WT-1* (*r*^2^ = .18), Supplementary Figure S2.

For individual patients, the mean correlation between GEP and RNA-seq for the five investigated genes is tight, i.e. a median correlation coefficient (Pearson) of 0.95 (see also Supplementary Figure S2 and discussion). For expression data regarding the functional validation cohort, see Table 1 and Supplementary Figure S1.

### Functional T-cell response

**Figure 2:**
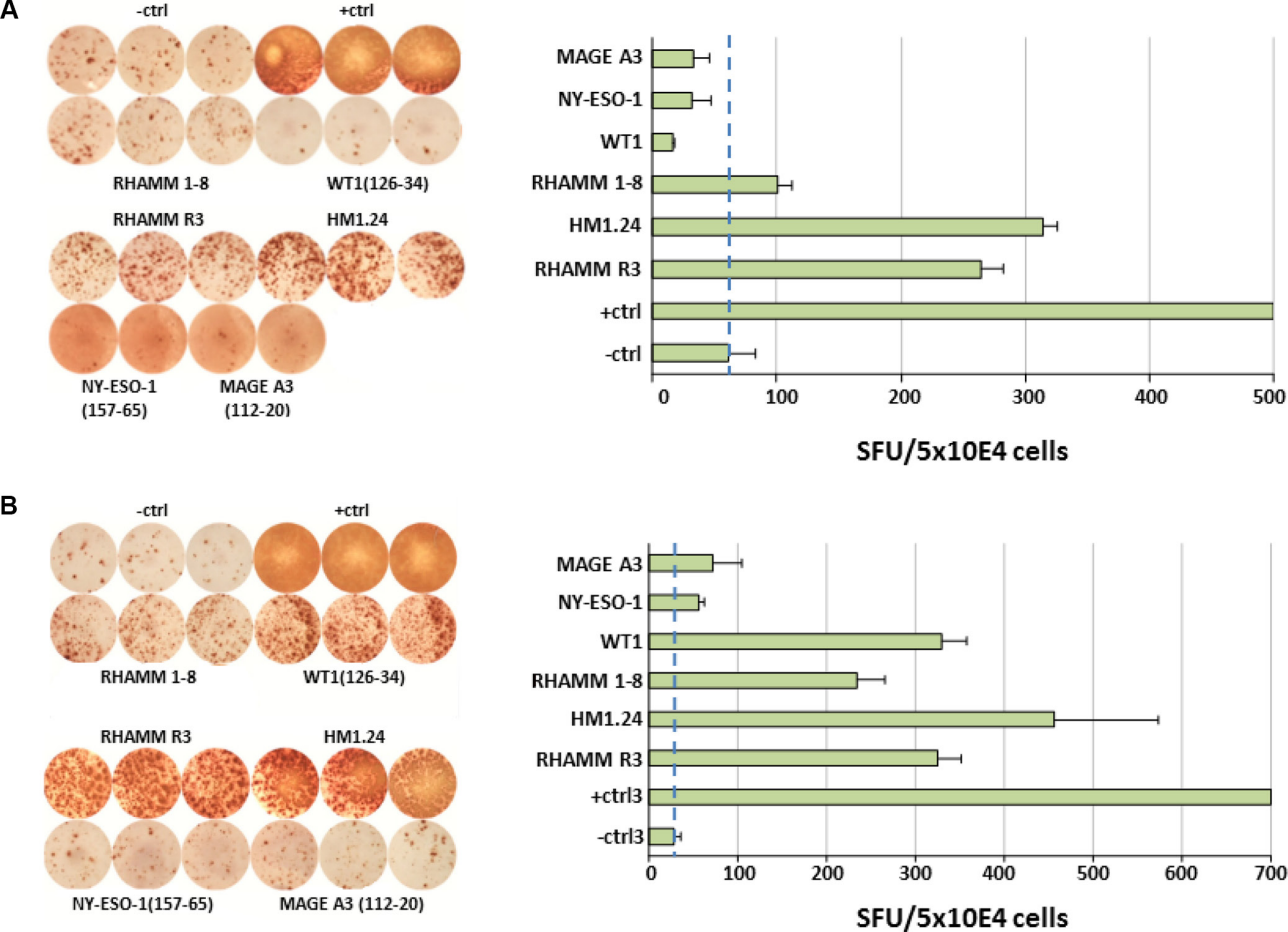
IFNγ-secretion of CTA-specific CD8^+^ T-cells from the bone marrow of patients with multiple myeloma Cells from myeloma patients were used in a MLPC with DCs as APC. In ELISPOT assay T2 cells served as APCs. In ELISPOTs, 5 × 10^4^ cells/well were seeded and analyses performed in triplicates. As negative control (-ctrl) no peptide or an irrelevant HIV-1 peptide was used. For positive control (+ctrl) SEB or CMVpp65 peptide were used. Dashed lines mark the background. Positive is 2 × SFU, strong positive 3 × SFU more than the negative control. (**A**) and (**B**) display exemplary results from 2/26 patients.

Regarding patients, as for any vaccination approach using HLA-A2-restricted peptides, applicability is limited to about half of patients, i.e. 53.6% (37/69) in our cohort (Supplementary Figure S3). In these, a T-cell response could be raised in 9/26 (34.6%) patients, of which 5/26 (19.2%) and 4/26 (15.4%) were classified as strong and weak, respectively. Regarding tests performed, a response was found in 19/80 tests (23.8%), i.e. 6/12 (50%) against HM1.24, 4/26 (15.4%) against HMMR3, 2/14 (14.3%) against HMMR1-8, 4/11 (36.4%) against WT-1, 1/9 (11.1%) against NY-ESO-1, and 2/8 (25%) against MAGE-A3. A strong response was found in a total of 10/80 (12.5%), i.e. 4/12 (25%) against HM1.24, 3/26(11.5%) against HMMR3, 2/14 (14.3%) against HMMR1-8, 1/11 (9.1%) against WT-1, as well as none of 9 and 8, respectively, against NY-ESO-1 or MAGE-A3 (Table 2). IFNγ secretion of CTA-specific CD8^+^ T-cells from the bone marrow of exemplary patients is shown in Figure 2.

**Table 2:** Overview of the amount of responses to the CTA-specific epitopes

Antigen	T-cell activation (all) in patients	T-cell activation (strong) in patients
	*n* (%)	*n* (%)
**HM1.24**	6/12 (50%)	4/12 (33%)
**HMMR-R3**	4/26 (15%)	3/26 (11%)
**HMMR1-8**	2/14 (14%)	2/14 (14%)
**MAGE-A3**	2/8 (25%)	0/8 (0%)
**NY-ESO-1**	1/9 (11%)	0/9 (0%)
**WT1**	4/11 (36%)	1/11 (9%)

Shown is a summary of the number of patients that recognize the different CTA-specific epitopes, respectively. The Empirical Rule applied here is 2 × SFU more than negative control. The percentage of the positive results in the groups of patients was calculated.

Of 10 observed strong T-cell responses in 22 patients with GEP-data available, 7 were observed with the respective antigen indicated as “expressed” vs. 3 in case of an “absent” signal (Table 1). Related to all tested antigens, responses were found in 12/35 tests where the respective antigen was expressed, vs. 7/26 in which this was not the case, (*P* = n.s.). In sample 4 showing a strong T-cell specific response, complete absence of *WT-1* expression was confirmed using RNA-sequencing (0 reads).

### Impact on survival

**Figure 3:**
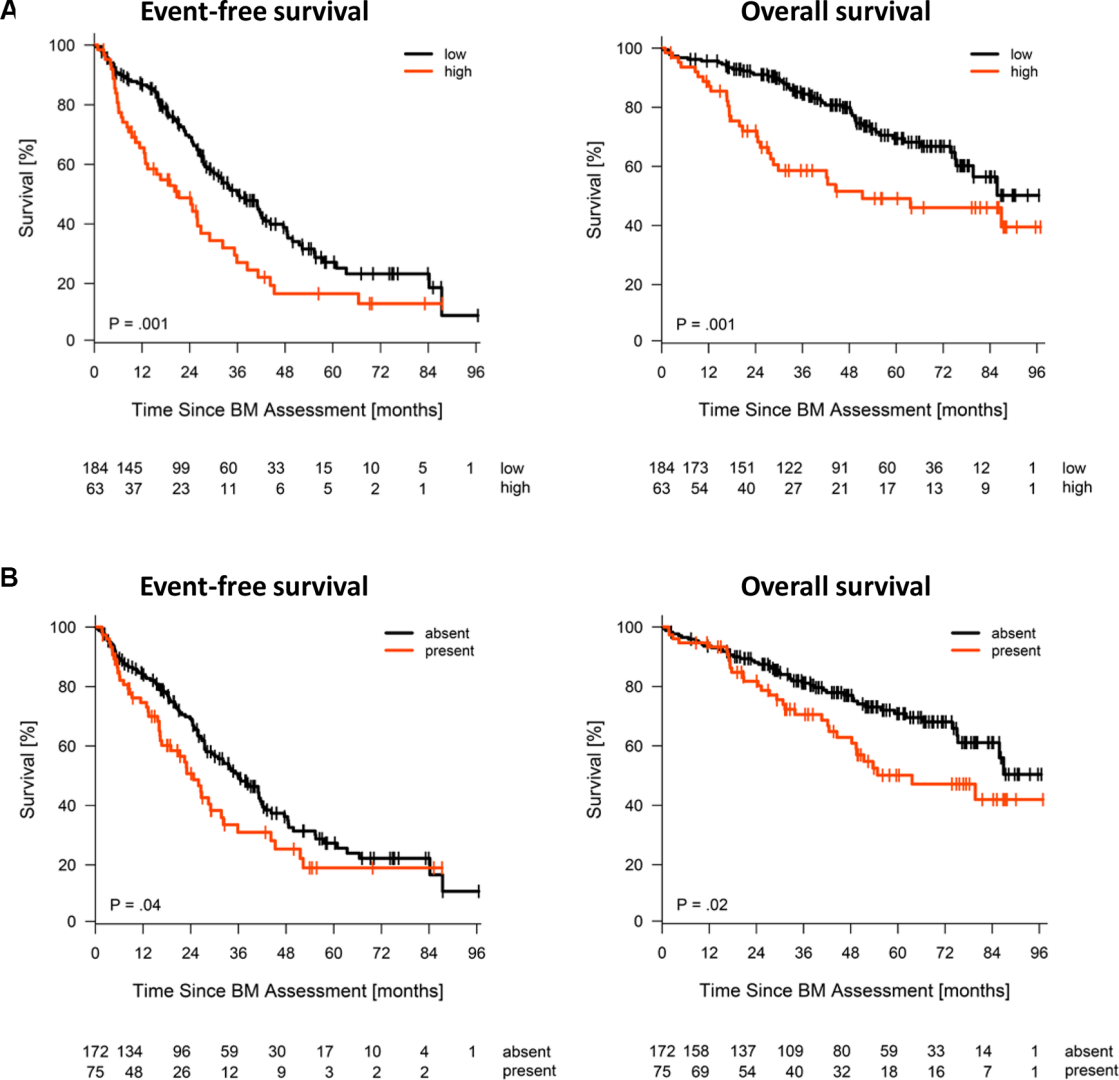

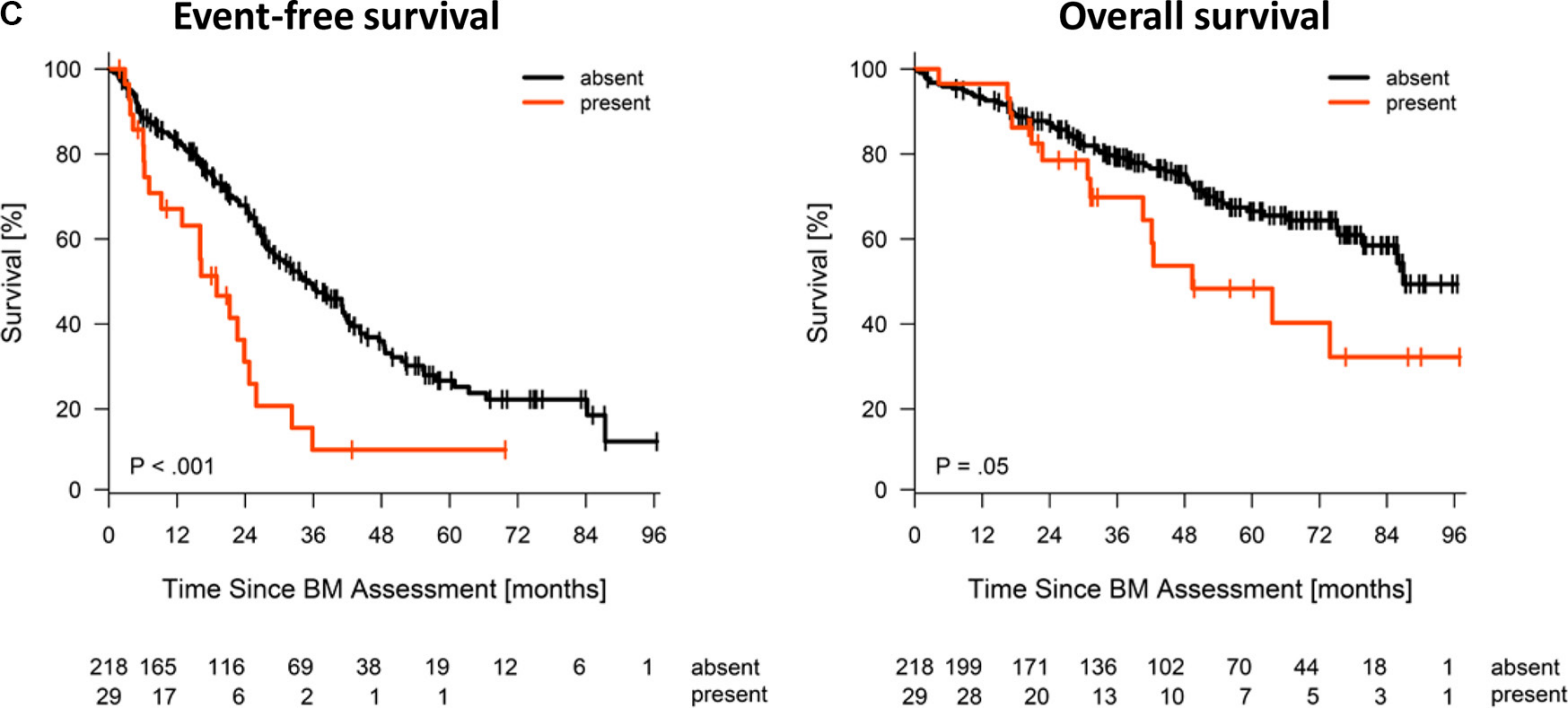
Expression of CTAs and survival in patients treated with high-dose therapy and autologous stem cell transplantation (*n* = 247) Depicted are event-free (EFS) and overall survival (OS) for (**A**) *HMMR*, (**B**) *MAGE-A3*, and (**C**) *NY-ESO1/2*. HMMR expression is grouped into “low” and “high” expression using maximally selected rank statistics for EFS and OS using the mean of the individual cut-offs for EFS and OS as cut-off. The PANP-algorithm is used to group *MAGE-A3* and *NY-ESO-1/2* expression in “present”, i.e. expressed, vs. “absent”, i.e. not expressed.

**Figure 4:**
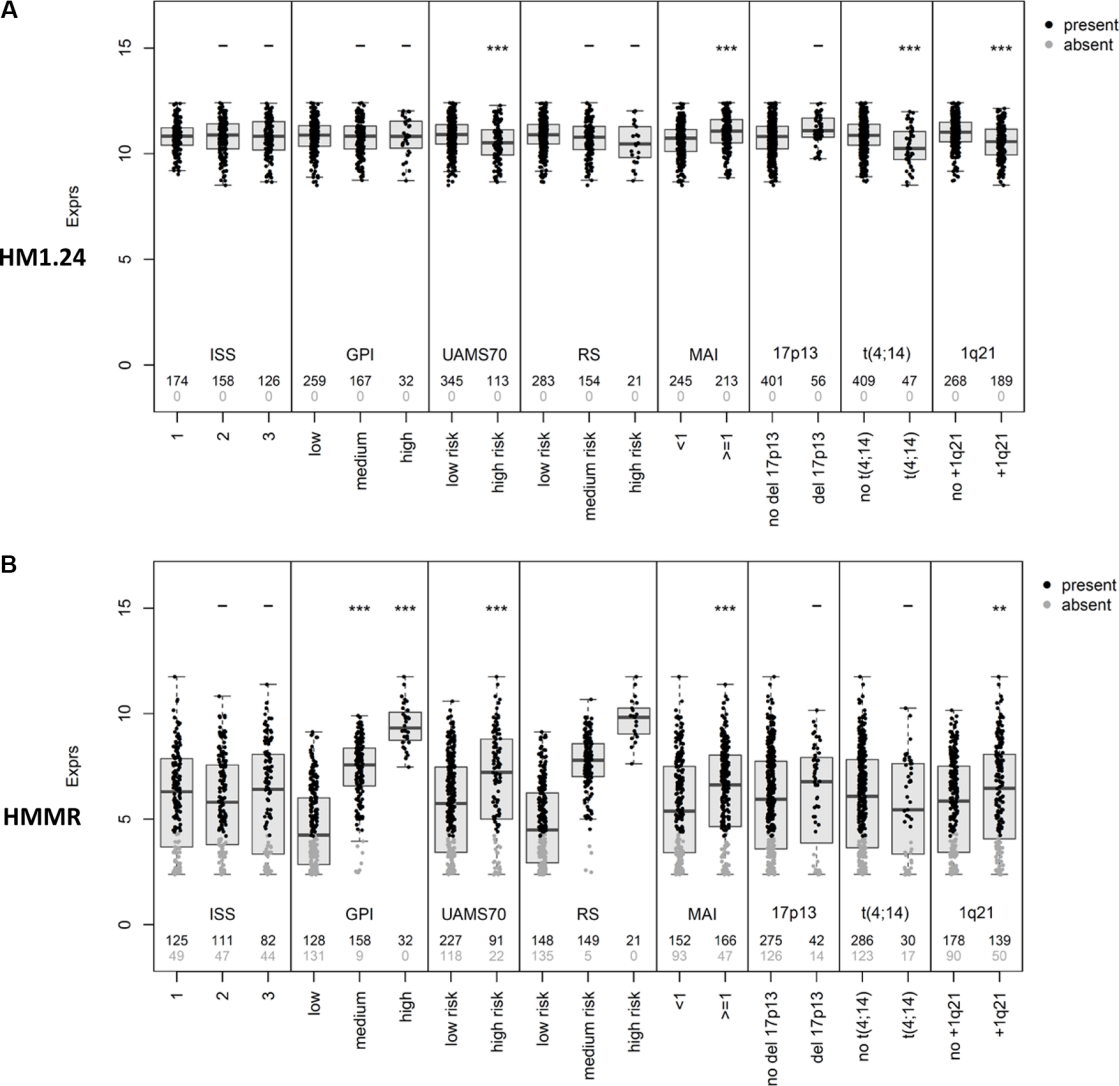

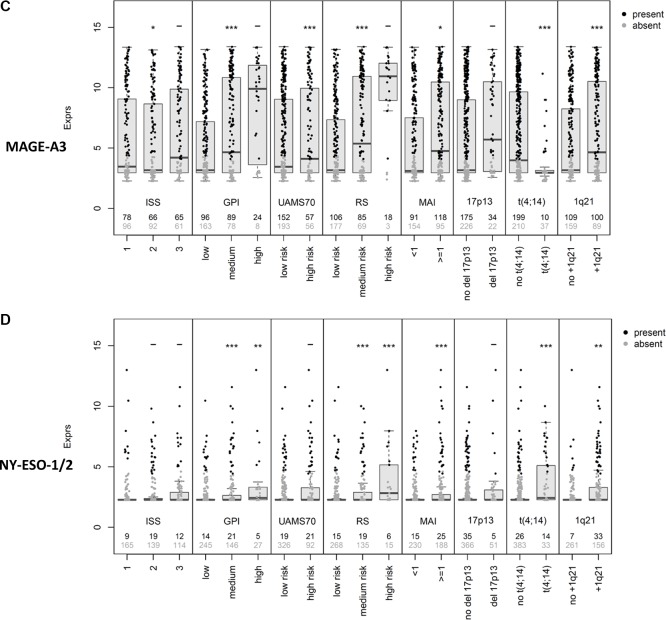

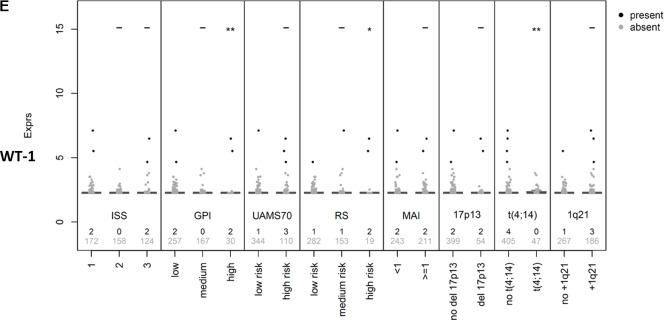
Correlation of CTA expression with tumor mass and molecular characteristics Shown is the correlation of CTA expression for (**A**) *HM1.24*, (**B**) *HMMR*, (**C**) *MAGE-A3,* (**D**) *NY-ESO-1/2*, and (**E**) *WT-1* with tumor mass (i.e. ISS stage [1 vs. 2 vs. 3]), proliferation (i.e. gene expression-based proliferation index, GPI [GPI^low^ vs. GPI^medium^ vs. GPI^high^]) and gene expression-based risk scores (i.e. UAMS70-gene score [low vs. high risk], Rs-score [low vs. medium vs. high risk], MYC activation index (MAI) [< 1 vs. ≥ 1]), as well as high-risk chromosomal aberrations according to interphase fluorescence *in situ* hybridization (i.e. presence of deletion 17p13, t(4;14), or gain 1q21), respectively. Significant differences between groups (Wilcoxon rank-sum test) are depicted by one asterisk (*) for a level of *P* < 0.05, two asterisks (**) for a level of *P* < 0.01, and three (***) for *P* < 0.001. As *HMMR* is part of the Rs-score, no statistical test was performed in this case.

**Figure 5:**
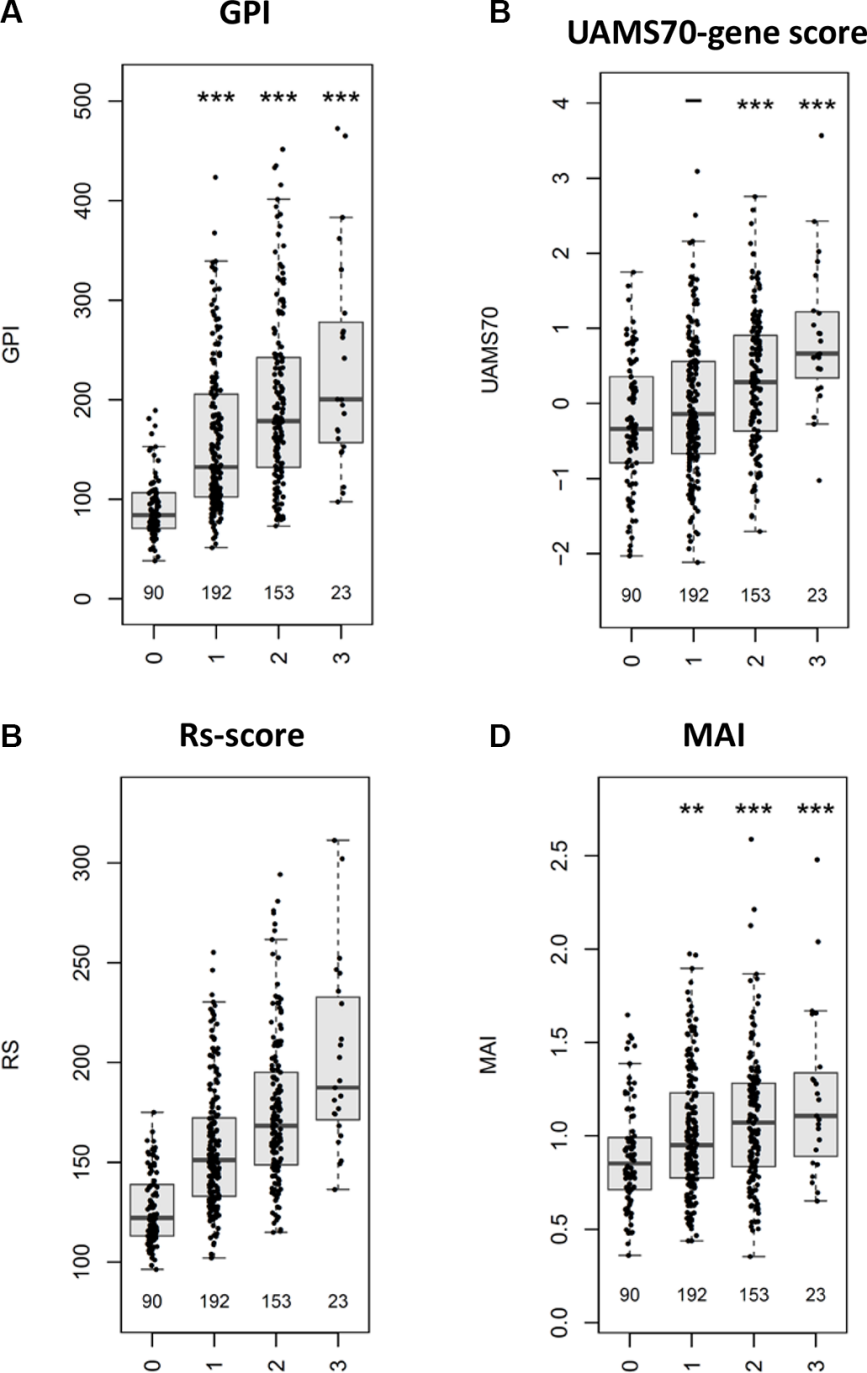
Association of the number of CTAs and risk-/proliferation-scores The number of the survival relevant CTAs *HMMR*, *MAGE-A3*, and *NY-ESO-1/2* (depicted on the x-axis) is significantly associated with gene expression-based risk-scores and proliferation (GPI) as shown for (**A**) GPI, (**B**) UAMS70-gene score, (**C**) Rs-score, and (**D**) MYC activation index (MAI). Using the Jonckheere-Terpstra trend-test, a significant trend (*P* < .001) can be found in all comparisons. Significant differences between groups (Wilcoxon rank-sum test) are depicted by one asterisk (*) for a level of *P* < 0.05, two asterisks (**) for a level of *P* < 0.01, and three (***) for *P* < 0.001. As *HMMR* is part of the Rs-score, no statistical test was performed in this case.

We next investigated an independent cohort of 247 patients treated by high-dose therapy and autologous stem cell transplantation with comparable expression pattern regarding the association of CTA expression with event-free and overall survival. Of the investigated antigens, *HMMR*, *MAGE-A3*, and *NY-ESO-1/2* are significantly associated with inferior event-free and overall survival (Figure 3). No association with survival is found for the constitutively expressed *HM1.24* as well as for *WT-1* (data not shown). For the correlation of CTA expression and tumor mass (i.e. ISS stage), gene expression-based risk-scores (i.e. UAMS70-gene score, Rs-score, MYC-activation index) and proliferation, as well as chromosomal aberrations (i.e. t(4;14) deletion 17p13, gain 1q21) see Figure 4. To answer the question if *HMMR*, *MAGE-A3*, and *NY-ESO-1/2* are independent, we first performed a multivariate analysis in which only *HMMR* remained significant (Supplementary Table S1). The strong prognostic impact of *HMMR* can be explained by gene expression being highly associated with proliferation of malignant plasma cells (Figure 4). The gene is likewise part of our gene expression-based risk score, i.e. Rs-score [37]. Secondly, we assessed the number of aberrantly expressed CTAs finding an association of the number of expressed CTAs > 1 and adverse event-free survival, but not overall survival (Jonckheere-Terpstra trend-test, *P* < .001; Supplementary Table S2). Again, the likely explanation of this is the association of the number of CTAs and risk-/proliferation-scores (Figure 5).

## DISCUSSION

### HM1.24 and CTAs as vaccinational targets in symptomatic myeloma

For clinical applicability in terms of potential vaccination trials, several conclusions can be drawn from our trial population based study. As for any vaccination approach using HLA-A2-restricted peptides, applicability is limited to about half of patients, i.e. 53.6% (37/69) in our cohort. In these patients, T-cell responses could be raised in 9/26 (34.6%) patients, leading to a patient population based rate of 18.4%. Even if the respective antigen was expressed (“mimicking” vaccination), e.g. *HM1.24* (in all), *HMMR* (in 69.4% of samples), only in about half of cases a T-cell response could be raised (Tables 1, 2, Figure 1, Supplementary Figure S1). With increasing numbers of CTAs used, T-cell responses become more likely (Table 2). Both findings suggest the use of a polyvalent vaccine, i.e. a “cocktail” of peptides as a vaccines, as previously envisioned by us based on CTA-expression alone [34]. Of the investigated antigens, *HM1.24* (100%), *HMMR* (64.9%), and *MAGE-A3* (45.6%) are frequently expressed targets. As is *NY-ESO-1/2*, with the caveat of delineation which of the three genes actually underlies the observed expression. Regarding GEP, this is due to the interrogation of all three genes (*CTAG1A*, *CTAG1B*, *CTAG2*) by probe set 210546_x_at, and *CTAG1A* as well as *CTAG1B* alongside a third and fourth probe set (2173339_x_at and 211674_x_at). Regarding RNA-sequencing, a potential difficulty is the observed high sequence homology in genes coding for *NY-ESO-1/2*, in turn leading to reads in RNA-sequencing simultaneously mapping to different genes, and, consecutively, are discarded due to “low quality” depending on the aligner and counting algorithm used. This potentially also explains the high variability in reported frequencies of NY-ESO-1 expression [24, 38, 39]. The reported frequencies here are however in agreement with detected frequencies of *NY-ESO-1* expression previously found by us using qRT-PCR [2].

Expression of CTAs is associated with adverse survival patients in treated by high-dose therapy and autologous stem cell transplantation (Figure 3), confirming earlier reports by others and us [34, 36]. This could be of special interest if, as e.g. shown for AML by us [31], likewise myeloma patients with HMMR-T-cell-responses would have better survival, and thus respective approaches especially impact on patients with more adverse risk. A further conclusion from the expression pattern of CTAs is that these would need to be measured within a clinical vaccination trial - raised T-cell responses would evidently only be useful if the target antigen is (or will be) expressed on the respective malignant plasma cells. As demonstrated here and in previous work by our group, gene expression (GEP-R) [35] or RNA-sequencing based reporting tools constitute convenient methods for such analyses.

### Association between antigen expression and specific t-cell responses

T-cell responses in absence of expression of the respective target on malignant plasma cells seem strange at first sight. However, it is unlikely that this is due to lack of sensitivity regarding detection. First, we had previously shown immune response against CTAs to be detectable despite absence of antigen expression in malignant plasma cells by quantitative real-time PCR [2]. We confirm this here using a different method (GEP) and the additional antigens HMMR and WT-1. In the latter, responses where exclusively detected with concomitant absent expression, validated with RNA-sequencing in exemplary patients from this cohort, indeed showing *no* respective reads e.g. patient 4 for *WT-1* despite T-cell activation. However, T-cell activation does not imply actual lysis of myeloma cells which prerequisites expression of the respective target antigen, as it detects the presence of a population of respective responsive T-cells only. Generation of reactive T-cells thus could have been against another -likely minor- population of (myeloma) cells present at a site different from the one from which the random aspirate for molecular analysis was drawn (spatial heterogeneity [40]), or represent cross reactivity between antigens (as e.g. for MART1/HM1.24) [41].

Lack of T-cell responses despite target expression is less difficult to understand as one of the most prominent effects of accumulation of malignant plasma cells in myeloma is induction of immunosuppression visible in dysfunctional DCs and diminished T-cell activation [2–4]. Potential circumvention of this effect is augmenting immune response by adding immunomodulatory drugs like IMiDs [42, 43], or using vaccination approaches at earlier stages e.g. asymptomatic myeloma or MGUS [2]. One of the remaining caveats (and limitation of our study) only answerable in a clinical vaccination trial is whether T-cell activation indeed transmits into lysis of myeloma cells expressing the respective antigen.

## MATERIALS AND METHODS

### Patients, healthy donors, and samples

Patients with newly diagnosed symptomatic myeloma (*n* = 458 with available GEP data, see below) according to CRAB criteria [44] and healthy donors (*n* = 20) were included in the study approved by the ethics committee (#229/2003, #S-152/2010) after written informed consent. Patients were treated in the prospective, randomized, open-label GMMG-MM5 phase III trial (EudraCT no. 2010-019173-16) [45]. The study is performed in accordance with the Declaration of Helsinki, the European Clinical Trial Directive and was approved by the local ethics committees of all participating institutions. See Supplementary Table S3 for patient characteristics.

The prognostic impact of CTA-expression was assessed on an independent cohort of 247 newly diagnosed, therapy-requiring patients treated with high-dose chemotherapy followed by autologous stem cell transplantation as published previously [46].

Normal bone marrow plasma cells and myeloma cells were purified using anti-CD138 microbeads (Miltenyi Biotec, Bergisch Gladbach, Germany) as published [46–49]. The negative fraction after plasma cell purification from *n* = 72 patients foreseen to be included in the GMMG-MM5 trial (including two screening failures) was frozen and used for the generation of DCs as described below (Supplementary Figure S3). The myeloma cell lines L363, SK-MM-2, LP-1, RPMI-8226, AMO-1, KMS-18, JIM-3, JJN3, KARPAS-620, KMS-12-BM, ANBL-6, KMS-11, MM1S, NCI-H929, KMS-12-PE, U266, OPM2, MOLP-8, MOLP-2, KMM-1, and EJM were purchased from the German Collection of Microorganisms and Cell Cultures, American Type Cell Culture, or Japan Health Science Research Resources Bank; the HG-lines HG1, HG3-HG9, HG11-HG15 and HG17 were generated in the Myeloma Research Laboratory Heidelberg (Germany), the XG-lines XG1-XG7, XG10-XG14, XG16, XG19-XG24 at INSERM (Montpellier, France) [50]. Peripheral blood CD27^+^ memory B-cells (*n* = 9) and polyclonal plasmablasts (*n* = 9) were generated as published [51, 52].

### Analysis of gene expression

Gene expression profiling was performed using U133 2.0 plus arrays (Affymetrix, Santa Clara, CA, USA) [47–49, 53]. Expression data are deposited in ArrayExpress under the accession numbers E-MTAB-2299, E-MTAB-317, E-TABM-937, and E-TABM-1088.

### RNA-sequencing

Full-length double-stranded cDNA was generated from 5 ng of total RNA and amplified using the SMARTer Ultra Low RNA Kit (Illumina, San Diego, CA, USA). Library preparation was performed from 10 ng of fragmented cDNA using the NEBNext Chip-Seq Library Prep protocol (New England BioLabs, Frankfurt am Main, Germany). Libraries were sequenced on an Illumina Hiseq2000 with 2 × 50-bp paired-end reads (*n* = 152).

### HLA typing

Cryopreserved cells were thawed and tested for the presence of HLA-A2 by flow cytometry using anti-human HLA-A2 antibody labeled with fluorescein isothiocyanate (FITC) (BB7.2, mouse IgG2b (κ)) (Biolegend, Fell, Germany). Flow cytometry was performed using a LSRII flow cytometer (BD Biosciences, Heidelberg, Germany) and analysis was done with FACSDiva Version 6.1.2 (BD Biosciences).

### Peptides

All HLA-A2-restricted CTA-derived peptides (for sequences see Table 3) were synthesized at our institution. SEB (staphylococcal enterotoxin B, Sigma-Aldrich, Darmstadt, Germany) and the HLA-A2 restricted CMV-pp65 epitope were used as positive controls. The HLA-A2 restricted HIV gag SL9 epitope and/or the no-peptide-control were used as negative control.

**Table 3:** Sequences of the HLA-A2-restriced CTA-specific peptides

Antigen	Sequence
**Melan-A/MART-1**	**ELAGIGILTV**
**HMMR-R3**	**ILSLELMKL**
**HMMR1-8**	**MSFPKAPL**
**MAGE A3**	**KVAELVHFL**
**NY-ESO-1 (CTAG1A)**	**SLLMWITQA**
**NY-ESO-1 (CTAG1B)**	**SLLMWITQA**
**NY-ESO-2 (CTAG2)**	**SLLMWITQA**
**WT-1**	**RMFPNAPYL**
CMV-pp65*	**NLVPMVATV**
HIV gag**	**SLYNTVATL**

Shown are the sequence and the amino acid positions of the used peptides, respectively. *CMV-pp65 served as a positive control. HMMR3 and HMMR1-8 are two different RHAMM/HMMR-derived T-cell epitope peptides as defined by others and us [32, 66]. ** HIV gag served as a negative control. We have previously shown the immunogenic oligo-peptide HM1.24aa22-30 (LLLGIGILV) as HLA-A2 restricted T-cell epitope (20) as well as cross-reactivity of HM1.24aa22-30 (LLLGIGILV) with the Melan-A/MART-1 derived peptide Melan-Aaa26-35*A27L (ELAGIGILTV) (21), indicated by their sequence conformity at the central peptide position (GIGIL).

### Generation of dendritic cells

Generation of DCs from HLA-A2^+^ patients was performed as described previously [54]. In brief, HLA-A2 positive tested patient cells were seeded at a density of 4 × 10^4^/well in a 6-well-plate in complete medium consisting of RPMI-1640 with 10% fetal bovine serum (FBS; both Thermo Fisher Scientific, Braunschweig, Germany) and cultured for 2 h at 37°C, and 5% CO_2_ to allow an enrichment of monocytes by plastic adherence. After aspirating the supernatant and washing twice with phosphate buffered saline (Sigma-Aldrich) the differentiation medium consisting of RPMI-1640 with 10% FBS, 500 U/ml interleukin-4 (IL-4, PeproTech, Hamburg, Germany) and 500 U/ml granulocyte macrophage colony-stimulating factor (GM-CSF, PeproTech) was added. After five days of culture the medium was completely changed and replaced by maturation medium containing a pro-inflammatory cytokine cocktail (RPMI-1640, 10% FBS supplemented with 1 µg/ml prostaglandin E [Sigma-Aldrich], 50 ng/ml tumor necrosis factor alpha [TNFα, Miltenyi Biotec], 10 ng/ml interleukin-6 [IL-6, PeproTech], and 10 ng/ml interleukin-1 β [Miltenyi Biotec]). Mature non-adherent DCs were harvested after two additional days of culturing and used as antigen presenting cells (APCs).

### Mixed lymphocyte peptide culture (MLPC)

The moDCs were pulsed for two hours with either a CTA-derived peptide or a control peptide at a concentration of 10 µg/ml. CD8+ T lymphocytes were selected from bone marrow mononuclear cells by magnetic-activated cell-sorting (Miltenyi Biotec). Following co-incubation of both at a ratio of 5:1 (moDc:CD8^+^; in 96-well plate 2 × 10^4^ moDc : 1 × 10^5^ CD8^+^) in complete medium, the culture was supplemented with 10 U/ml IL-2 and 20 ng/ml IL-7 (both from PeproTech) on day +1. On day seven cells were washed once with medium and subsequently used for interferon-γ (IFNγ) ELISPOT (Enzyme Linked Immuno Spot) assays (see below).

### Interferon-γ ELISPOT assay

IFNγ ELISPOT assays were performed as described earlier [31, 32, 55]. In brief, 96-well hydrophobic IP Multiscreen Filter Plates (Merck Millipore, Darmstadt, Germany) were activated with 35% ethanol and coated with anti-human IFNγ antibody (Mabtech, Nacka Strand, Sweden) overnight at 4°C. The next day plates were washed with phosphate buffered saline and blocked with 10% FBS for 2 h at room temperature. Presensitized CD8^+^ cells (effector cells) from MLPC day seven were co-incubated with peptide pulsed T2 cells at a ratio of 1:1 in triplicates (5 × 10^4^ effectors per well) for 20 h. The secretion of IFNγ was detected using biotinylated anti-human IFNγ antibody (Mabtech), Streptavidin HRP and HRP substrate set (BD Biosciences) according to the manufacturer’s instructions. The spot forming units (SFU) were counted and analyzed using a CTL-ImmunoSpot^®^ analyzer equipped with the ImmunoSpot 5.0.9 software (CTL, Bonn, Germany).

A specific T-cell response against the respective antigen was defined as ≥ 2-fold increased number of positive ELIspots compared to control, a strong response was defined as ≥ 3-fold increased number of positive ELIspots compared to control.

### Statistical analysis

### RNA-sequencing

Next generation sequencing RNA fastq-files were aligned with STAR aligner [56] to the GRCh38 reference genome (http://www.ensembl.org/Homo_sapiens/Info/Index). Reads were counted with the summarizeOverlaps function from the Bioconductor package GenomicAlignments [57]. This function was used without quality filtering, as most *CTAG1A, -B*, and *CTAG2* reads mapped to multiple positions due to high sequence homology. Technical replicates were summed and subsequently normalized with the counts-per-million function of edgeR [58]. For depicting a gene as “expressed” in RNA-sequencing, a conservative threshold of > 1 normalized read count was used. This corresponds to 22.7 ±- 13.3 reads supporting the expression of the respective gene. The percentage of patients fulfilling this criterion can be interpreted as lower limit of the percentage of patients expressing the respective gene. Expression profiles of 152 symptomatic multiple myeloma patients, 19 myeloma cell lines, 10 normal bone marrow plasma cell, 4 B-memory cell, and 4 polyclonal plasmablastic cell samples were analyzed. Expression values are depicted as ln (normalized counts +1).

### Gene expression profiling

Microarray gene expression analyses were performed on GC-RMA [59] preprocessed data sets of the B-cell lineage. To assess the presence or absence of gene expression the “Presence-Absence calls with negative Probesets” algorithm (PANP) was used [60].

Gene expression-based assessment of risk (UAMS70-gene score [61], Rs-score [37], and MYC activation index (MAI) [62]) and proliferation [53] were performed as previously published. For calculation of the UAMS70-gene score and MAI, the cohort was normalized with the mas5 algorithm.

### Survival and further analyses

Event-free and overall survival were assessed using the Kaplan-Meier method [63]. Differences between curves were tested with Log-Rank test. HMMR expression is grouped into “low” and “high” expression using maximally selected rank statistics for event-free and overall survival using as cut-off the mean of the individual cut-offs for event-free and overall survival. The PANP-algorithm is used to group *NY-ESO-1* and *MAGE-A3* expression in “present” vs. “absent” (see above). Differences in gene expression between defined groups were investigated by Wilcoxon rank sum test.

Pearson’s correlation coefficient was calculated for comparison of microarray and RNA-seq gene expression.

The mean correlation coefficient was calculated using the Fisher-Z transformation transforming correlation coefficients, calculating the mean and subsequent back-transforming.

Statistical computations were performed using R [64], version 3.2.4 and Bioconductor, version 3.1 [65]. Effects were considered statistically significant if the *P*-value of corresponding statistical tests was below 5%.

## CONCLUSIONS

T-cell responses can –despite described immunosuppression– be raised frequently within the study population of our GMMG-MM5 trial, and vaccination approaches thus represent a therapeutic option. Expression of individual CTAs in a subfraction of patients only, and in turn generation of T-cell responses in an even smaller subfraction of these suggest simultaneous use of a cocktail of peptide vaccines. HM1.24, HMMR, MAGE-A3 and NY-ESO-1/2 are good candidates; the latter three associated with adverse prognosis. Secondly, it implies that CTA-expression in individual patients needs to be assessed in clinical vaccination trials.

## SUPPLEMENTARY MATERIALS FIGURES AND TABLES


