# Supplementary material for: Frequency of expression and generation of T-cell responses against antigens on multiple myeloma cells in patients included in the GMMG-MM5 trial
Source: Oncotarget. 2016 Aug 11;8(49):84847–62. doi: 10.18632/oncotarget.11215 (PMC5689578; doi:10.18632/oncotarget.11215)
Supplement: Supplementary file 1 [file oncotarget-08-84847-s001.pdf]

Frequency of expression and generation of T-cell responses against antigens on multiple myeloma cells in patients included in the GMMG-MM5 trial

Supplementary Materials

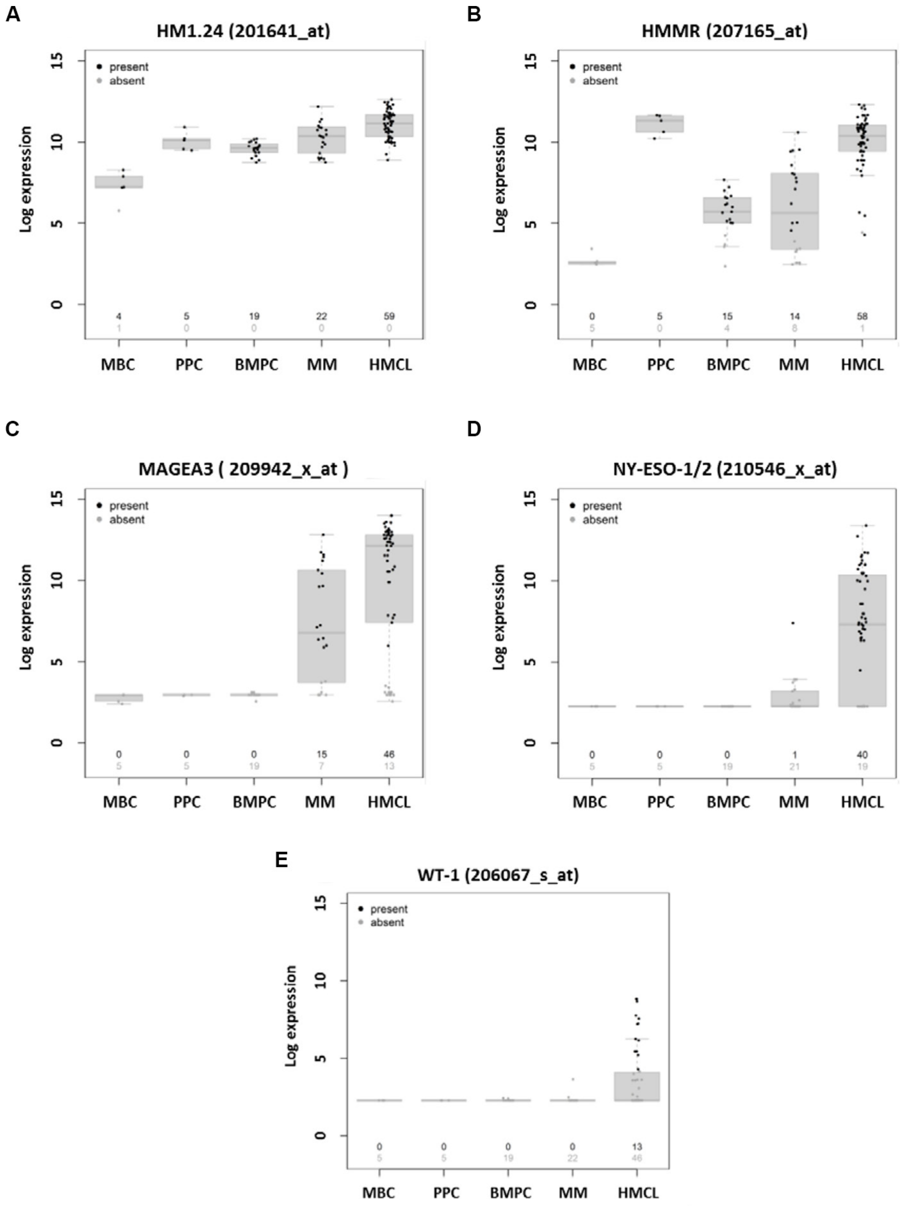

Supplementary Figure S1: Expression of tumor associated antigens in normal and malignant plasma cells as well as cells of the B-cell lineage (functional validation cohort). Shown is the expression of (**A**) *HM1.24*, (**B**) *HMMR*, (**C**) *MAGE-A3*, (**D**) *NY-ESO-1*, and (**E**) *WT-1* in normal plasma cell precursors, i.e. memory B-cells (MBC) and *in vitro* generated polyclonal plasmablastic cells (PPC), as well as normal bone marrow plasma cells (BMPC), malignant plasma cells from patients with newly-diagnosed multiple myeloma (MM; functional validation cohort, only), and human myeloma cell lines (HMCL). For myeloma patients, analysis is restricted to patients assayed for T-cell response and for whom gene expression data as assessed by DNA-microarrays were available. Black numbers depicting the number of patient samples expressing the respective gene according to the PANP-algorithm, gray number the one that showed no expression. *HM1.24* is expressed in all of 22 samples for which GEP-data were available, *HMMR* in 14/22 (63.6%), MAGE-A3 in 15/22 (68.2%), *NY-ESO-1* in 1/22 (4.5%), and *WT-1* in 0/22 (0%). Using RNA-sequencing, *HM1.24* and *HMMR* are expressed in all of seven samples with data available, *MAGE-A3* in 6/7 (86%), *NY-ESO-1/2* in 3/7 (43%), and *WT-1* in 4/7 (57%).

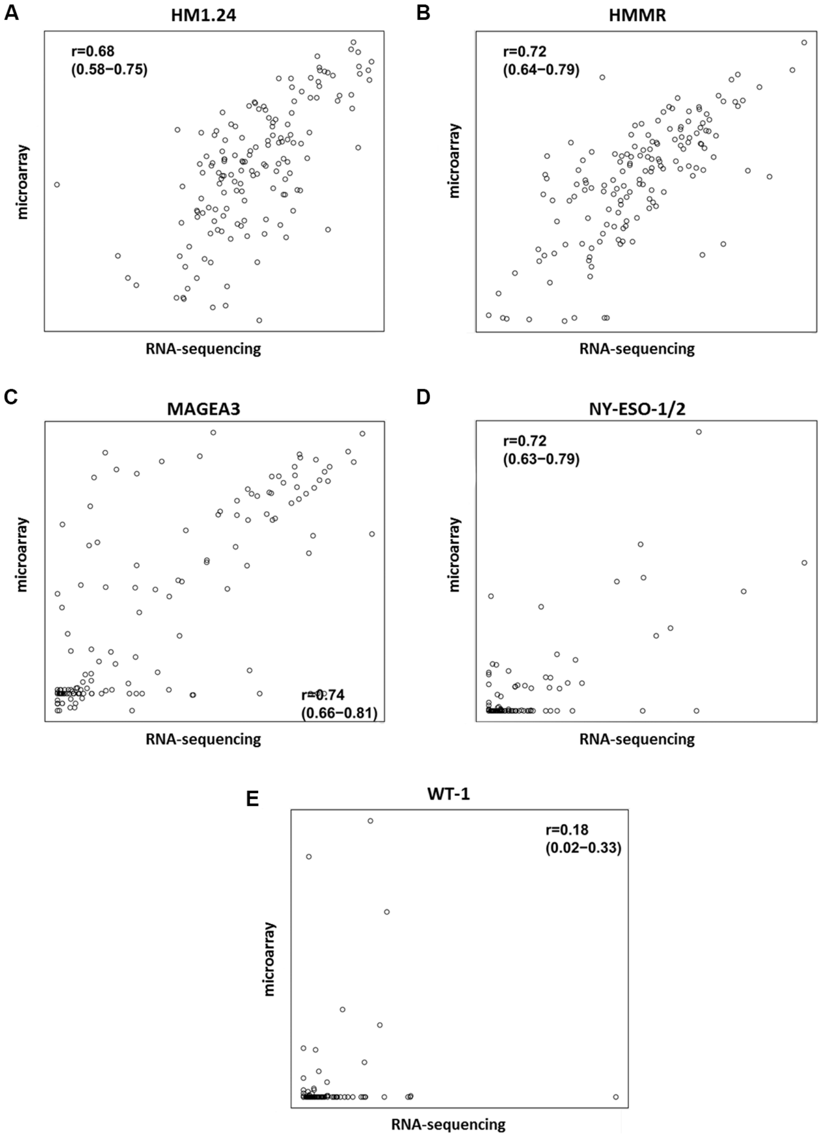

Supplementary Figure S2: Correlation plot. Expression based on DNA-microarray is plotted vs. RNA-sequencing for (**A**) *HM1.24*, (**B**) *HMMR*, (**C**) *MAGE-A3*, (**D**) *NY-ESO-1/2*, and (**E**) *WT-1*. Pearson correlation coefficient (r) with corresponding 95% confidence interval is shown for the respective CTAs.

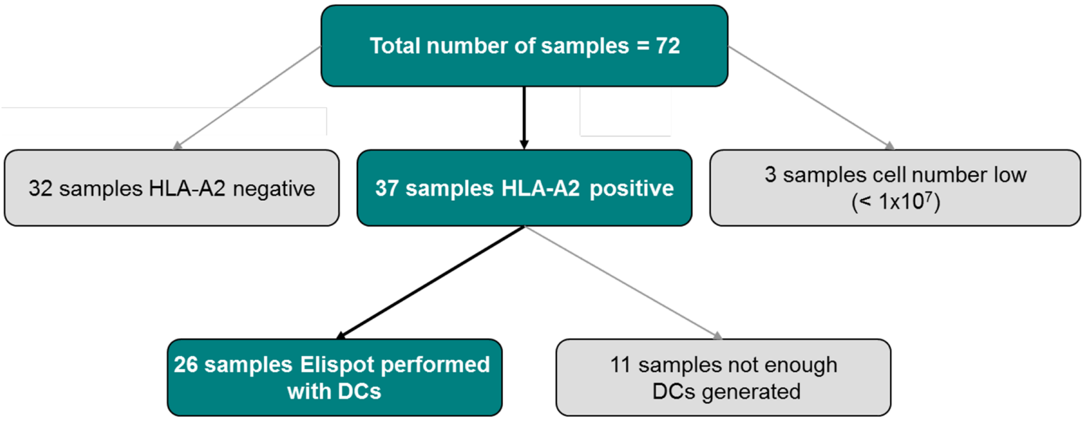

Supplementary Figure S3: Samples for functional testing. From a total of 72 consecutive samples from patients to be included in the GMMG-MM5 trial, 69 could be used for further testing regarding available number of cells within the CD138-negative fraction. Of these, 37/69 (53.6%) were HLA-A2 positive and could be used for further testing. Of the latter, for 26 a sufficient number of DCs could be generated to perform ELISPOT-assay(s), see Table 1.

Supplementary Table S1: Multivariate analysis

Variable	HR	CI	*P* - value
**Event-free survival**			
HMMR low vs. high	0.66	0.5–1	**0.038**
MAGE-A3 absent vs. present	1.13	0.7–1.7	0.065
NY-ESO-1/2 absent vs. present	1.73	1.0–3.1	0.582
**Overall survival**			
HMMR low vs. high	0.52	0.3–0.8	**0.007**
MAGE-A3 absent vs. present	1.49	0.9–2.5	0.132
NY-ESO-1/2 absent vs. present	1.1	0.6–2.2	0.791

HR, hazard ratio. CI, confidence interval. HMMR expression is grouped into "low" and "high" expression using maximally selected rank statistics for event-free and overall survival using the mean of the individual cut-offs for event-free survival and overall survival as cut-off. The PANP-algorithm is used to group MAGE-A3 and NY-ESO-1/2 expression in "present", i.e. expressed, vs. "absent", i.e. not expressed. See Figure 3.

Supplementary Table S2: Univariate analysis for the number of expressed and survival relevant ctas, I.E. hmmr, mage-A3, and ny-Eso-1/2. hr, hazard ratio. ci, confidence interval

Variable	Number [*n*=]	Events [*n*=]	HR	CI	*P*-value
**Event-free survival**					
Number of expressed CTAs = 0	38	19			
Number of expressed CTAs = 1	135	79	1.13	0.7–1.9	0.633
Number of expressed CTAs = 2	51	31	1.83	1–3.2	**0.040**
Number of expressed CTAs = 3	23	16	2.45	1.3–4.8	**0.009**
**Overall survival**					
Number of expressed CTAs = 0	38	9			
Number of expressed CTAs = 1	135	38	1.08	0.5–2.2	0.844
Number of expressed CTAs = 2	51	21	1.96	0.9–4.3	0.091
Number of expressed CTAs = 3	23	12	2.21	0.9–5.3	0.073

Supplementary Table S3: Patient characteristics

Variable	Level	GEP cohort [*n* = 458]		ELISPOT cohort [*n* = 26]	
		n	**%**	***n***	**%**
Sex	male	273	59.6%	19	73.1%
	female	185	40.4%	7	26.9%
Age [years]	≤ 60	230	50.2%	14	53.8%
	> 60	195	42.6%	12	46.2%
	NA*	33	7.2%	0	0.0%
Type	IgA	91	19.9%	6	23.1%
	IgG	269	58.7%	18	69.2%
	IgD	1	0.2%	0	0.0%
	Bence Jones	97	21.2%	2	7.7%
Light chain type	Kappa	309	67.5%	15	57.7%
	Lambda	149	32.5%	11	42.3%
ISS stage	1	174	38.0%	10	38.5%
	2	158	34.5%	6	23.1%
	3	126	27.5%	10	38.5%

Shown are the characteristics for the whole GMMG-MM5 cohort with available GEP data for the assessment of CTA expression (n = 458 in total) as well as the cohort for whom ELISPOT assays were performed (n = 26). NA, not available. ISS, international staging system. *Age is not available in all cases as for some patients only the year of birth is available due to restrictions of the local ethics committees.
